# Rosai-Dorfman Disease as Constrictive Pericarditis: An Unusual Childhood Presentation

**DOI:** 10.7759/cureus.62062

**Published:** 2024-06-10

**Authors:** Kapil Dev Rabha, Himesh Barman, Shakthi A Kumar, Jaya Mishra, Reuben L Kynta, Donboklang Lynser, Pranjal Kalita

**Affiliations:** 1 Pediatrics and Neonatology, North Eastern Indira Gandhi Regional Institute of Health and Medical Sciences, Shillong, IND; 2 Pediatrics, North Eastern Indira Gandhi Regional Institute of Health and Medical Sciences, Shillong, IND; 3 Pathology, North Eastern Indira Gandhi Regional Institute of Health and Medical Sciences, Shillong, IND; 4 Cardiothoracic and Vascular Surgery, North Eastern Indira Gandhi Regional Institute of Health and Medical Sciences, Shillong, IND; 5 Radiology, North Eastern Indira Gandhi Regional Institute of Health and Medical Sciences, Shillong, IND

**Keywords:** lymphadenopathy, histiocytosis, pleural effusion, effusive-constrictive pericarditis, rosai-dorfman disease

## Abstract

Rosai-Dorfman disease (RDD) is a rare, multisystemic, histiocytic disorder that usually presents with painless cervical lymphadenopathy. We describe a case of constrictive effusive pericarditis with congestive cardiac failure in a six-year-old child as an initial presentation of RDD. The child underwent pericardiectomy and was treated with steroids, following which the symptoms resolved entirely. While tuberculosis accounts for the majority of cases presenting with constrictive pericarditis in developing countries, the presentation of RDD can be easily missed if not kept in mind as a possibility. This case report documents the unique presentation of a rare disease.

## Introduction

Rosai-Dorfman disease (RDD) is a histiocytic disorder of unknown etiology characterized by massive lymphadenopathy and the histological hallmark finding of the presence of an intact cell within the cytoplasm of another cell, also known as emperipolesis, with positive immunohistochemical staining for S100 and CD68 but negative for CD1a [1]. According to the revised classification of histiocytosis and neoplasms of macrophage-dendritic cell lineages by the Histiocyte Society, RDD is classified under histiocytosis of the R-group [2]. RDD principally affects children and young adults, with a mean age at onset of 21 years and a relatively male predominance [3]. The classic presentation of this disease is bilateral, massive, painless cervical lymphadenopathy, which can be associated with fever and night sweats. However, it can have a varied presentation based on the extranodal sites involved. Cardiac involvement is rare and is seen in 0.1-0.2% of RDD cases, with the majority of cases presenting as intracardiac mass [4]. Constrictive pericarditis as an initial presentation is rarely noted in adult RDD patients and is even rarer in pediatric patients [5,6]. Clinical findings along with radiological, histomorphological, and immunohistochemistry findings play a pivotal role in the diagnosis of such cases.

Here, we present the case of a child with cardiac RDD (cRDD) who presented with constrictive pericarditis and posed a diagnostic challenge.

## Case presentation

A six-year-old boy presented to the emergency department with complaints of fever, breathing difficulty, chest pain, and distension of the abdomen for about a year with gradual progression. There was no history of contact with tuberculosis or any significant family history. On presentation, the child had tachycardia, tachypnea with oxygen saturation of 90% in room air, raised jugular venous pressure, and no lymphadenopathy. On systemic examination, dull percussion notes were heard in the bilateral basal lung fields with decreased breath sounds, suggesting pleural effusion. The cardiovascular examination revealed soft heart sounds and no murmur or any other added sound. The child also had gross ascites and tender hepatomegaly. Blood workup showed normal hemoglobin for age (12.2 g/dL), thrombocytosis (700 × 10^3^/μL), elevated erythrocyte sedimentation rate (ESR) (27 mm at one hour), and hypoalbuminemia. There was no leucocytosis (9.6 × 10^3^/μL) or eosinophilia. Serum ferritin, interleukin 6, C-reactive protein (CRP), and procalcitonin were within the normal limit. Mantoux tuberculin skin test and cartridge-based nucleic acid amplification test (CBNAAT) for tuberculosis in gastric lavage were negative. Chest radiograph showed bilateral pleural effusion and cardiomegaly, suggestive of pericardial effusion with no other significant findings. Pleural fluid analysis revealed total counts of 140 cells/mm^3^, with a lymphocytic predominance of 60%. Glucose level was 101 mg/dL and protein was 0.3 gm/dL. The pleural fluid protein and serum protein ratio was 0.15, suggesting transudative effusion. Pleural fluid CBNAAT was negative, and no acid-fast bacilli were seen.

**Figure 1:**
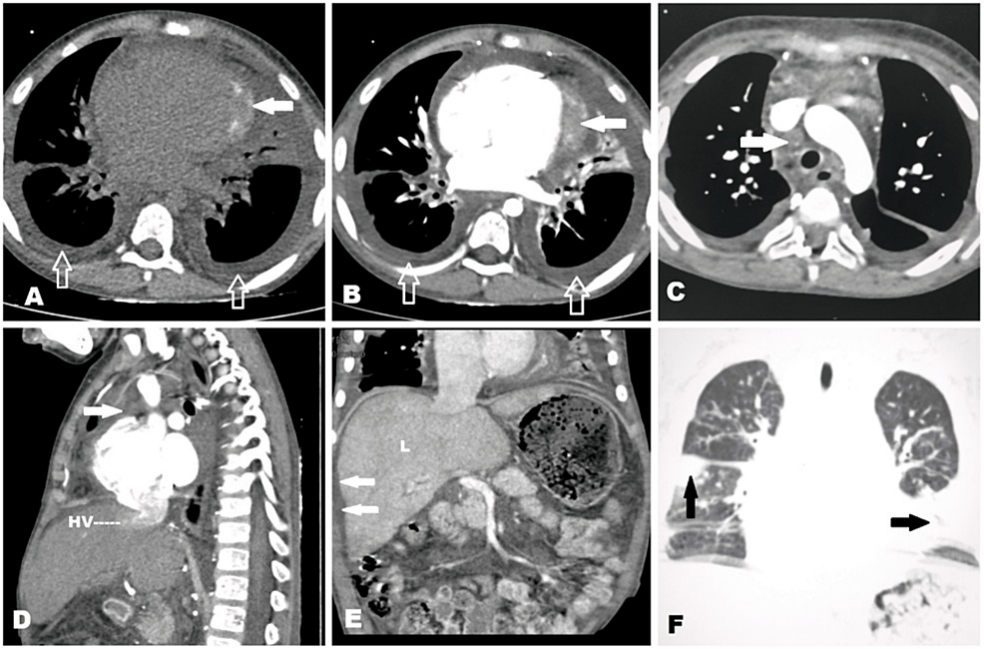
(A) Plain CT of the chest in the axial section showing a thickened pericardium with calcifications (solid white arrow) and bilateral pleural effusion (open arrow). (B) Contrast-enhanced CT in the axial section showing non-enhancing pericardial thickening (solid arrow) with bilateral pleural effusion (open arrow). (C) Contrast-enhanced CT in the axial section showing an enlarged mediastinal node (solid white arrow). (D) Contrast-enhanced CT in the sagittal section showing reflux of the contrast into the hepatic vein (HV) with enlarged mediastinal node (solid white arrow). (E) Coronal reconstructed contrast-enhanced CT of the abdomen showing minimal ascites (solid white arrow) with hepatomegaly (L). (F) Coronal reconstructed CT of the chest in lung window showing consolidation in both lung fields (black arrow).

Two-dimensional echocardiography showed biatrial enlargement, right atrial spontaneous echo contrast, with good left ventricular function and respiratory variation in mitral inflow pattern of more than 25%, suggestive of constrictive pericarditis. Contrast-enhanced CT of the thorax and abdomen was done to further characterize the condition. It showed pericardial thickening with calcification along the left ventricle with retrograde flow of contrast into the inferior vena cava, suggestive of constrictive pericarditis (Figure 1). It also showed bilateral pleural effusion, patchy consolidation in bilateral lungs, and mediastinal as well as hilar lymphadenopathy.

**Figure 2:**
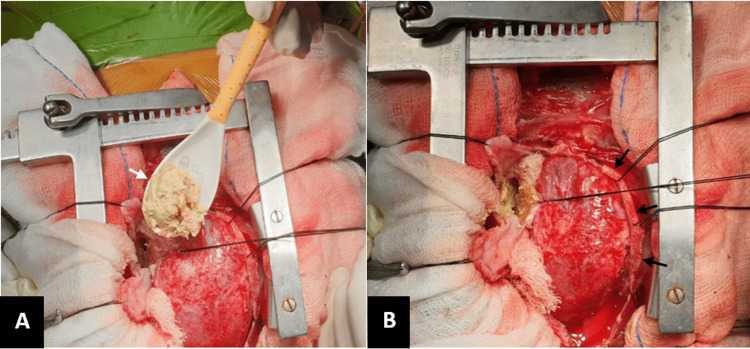
Intraoperative images showing (A) cheesy material (white arrow) and fibrinous exudates at the cardiac apex and (B) a thickened pericardium (black arrows).

The child was taken up for emergency pericardiectomy, and, intraoperatively, the pericardium was found to be thickened around 3 mm with adherence to the cardiac surface and great vessels restricting cardiac movement (Figure 2).

**Figure 3:**
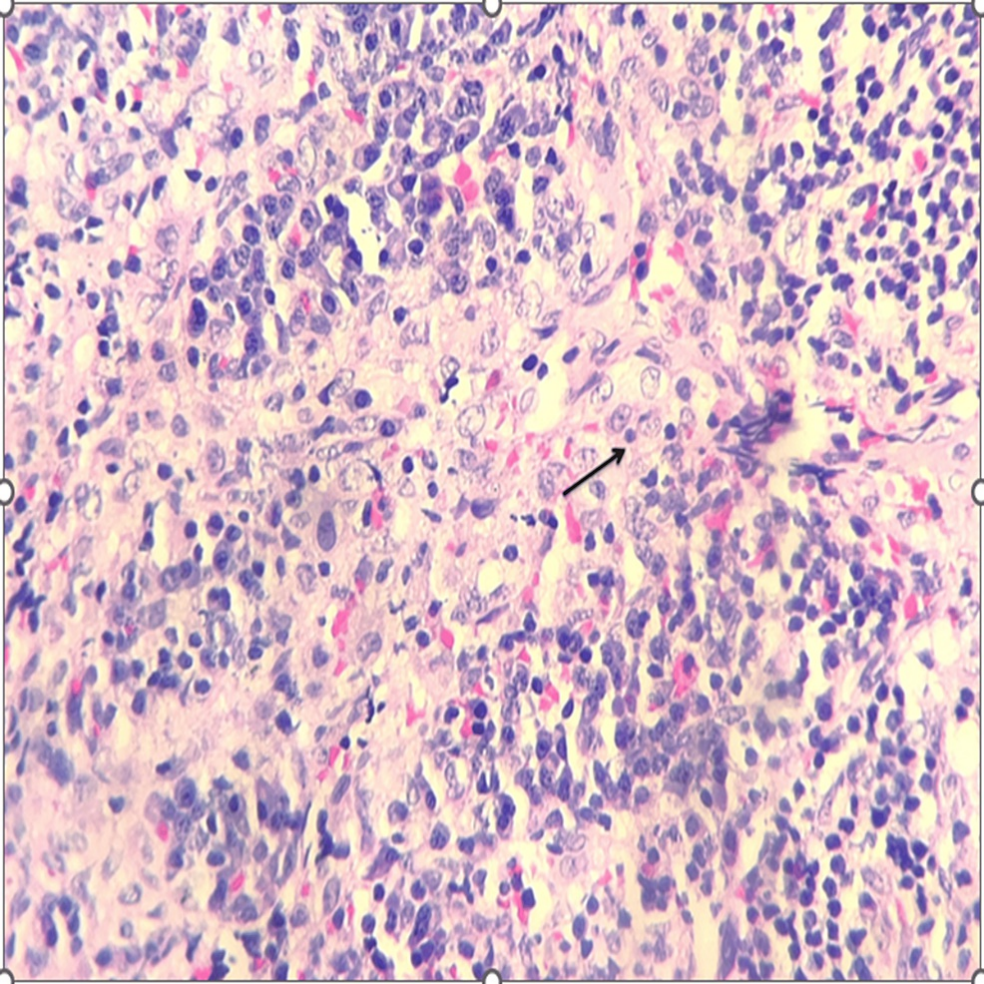
Histomorphological image showing the characteristic histiocytic collection along with inflammatory cell infiltrates consisting of lymphocytes and plasma cells. The histiocytes show round-to-oval nuclei with prominent nucleolus and a moderate amount of eosinophilic cytoplasm with evidence of emperipolesis (black arrow) (hematoxylin and eosin, 400×).

Biopsy samples were collected from the mediastinum, pericardium, as well as the pericardiac and para-aortic nodes. Histopathological examination of the pericardial and para-aortic nodes showed sinus histiocytosis with emperipolesis, a phenomenon that clinched the diagnosis of RDD (Figure 3). Immunohistochemical staining from the tissue samples was positive for S100 and CD68 and negative for CD1a immunostain.

The postoperative course was uneventful, and the child was started on prednisolone at a dose of 1 mg/kg/day for six weeks and then tapered off. The pleural effusion, along with the ascites, gradually resolved. Review echocardiography scans after six weeks revealed no pericardial effusion and good left ventricular function. The patient was asymptomatic at subsequent visits three months post-surgery and was advised to monthly review or report at the earliest if any symptoms arose.

## Discussion

RDD is a rare form of histiocytic disorder, presenting commonly as rapidly progressive painless lymphadenopathy. Although primarily nodal, extranodal RDD of the central nervous system, respiratory system, soft tissues and bones, urogenital system, and oral cavity is noted in the literature. The extranodal involvement of this disease is observed in approximately 43% of the cases. According to Giri et al., the involvement of the axillary, inguinal, and mediastinal regions is 23.7%, 25.7%, and 14.5%, respectively. Among the clinical symptoms, fever and weight loss are widely noted in patients of RDD; however, it is not entirely specific and symptoms are mainly site-specific [7]. Our patient had a rare presentation of RDD as constrictive pericarditis which posed a diagnostic challenge.

Massive, painless cervical lymphadenopathy is the most common presenting feature of the disease; however, in our patient, there was no palpable superficial lymph node. Routine laboratory investigations include complete blood count, ESR, and CRP. Increased ESR, increased total white blood cell count, normocytic normochromic anemia, neutrophilia, thrombocytosis, and CRP are usually noted in the literature for RDD patients. In our case, the patient presented with thrombocytosis and raised ESR but other parameters were within normal limits, showing that although routine laboratory investigations are helpful, they are not absolute for a diagnosis of RDD. Among the radiological investigations, ultrasonography serves as a relatively inexpensive and helpful modality for the identification of lesions presenting with lymphadenopathy; however, positron emission tomography-computed tomography, if available, has an important role in evaluating disease extent. Pediatric cRDD is uncommon compared to adults and has never been reported in the first decade of life. The most common mode of presentation appears to be with an intracardiac mass, followed by a pulmonary arterial mass, and, rarely, pericardial effusion. While the pleural or pericardial fluid analysis does not provide any direct diagnostic utility, it is important in ruling out various other etiologies such as tuberculosis and lymphomatous involvement of such spaces. Hence, if possible, it is recommended to be performed in the initial diagnostic workup differential diagnosis. Pericardial involvement in RDD has been reported only in adults, either in the form of pericardial mass, effusion, or infiltration [4].

The striking feature in our patient was that nodal RDD of pericardial and para-aortic nodes presented without superficial lymphadenopathy. Presenting as systemic cRDD due to infiltration of the pericardium is very rare in the literature. According to a literature review by O’Gallagher et al., three case reports of cRDD were found in the pediatric age group (less than 18 years of age). Among the three reported cases of pediatric cRDD, all were above 12 years of age [4]. Two patients had sickle cell disease and a similar presenting feature in the form of an intracardiac mass arising from the interatrial septum, associated with infiltration in other parts of the heart. Both had conduction abnormalities and required permanent pacing of the heart [8,9]. The other case involved tricuspid and pulmonary valves along with multiple extranodal site involvement, which was diagnosed during an autopsy [10]. Our patient presented with constrictive pericarditis, which resulted in marked systemic venous congestion. The absence of superficial lymph node involvement further masked the diagnosis.

The histopathological findings in this disease are similar in all involved locations. The histiocytes in RDD are large, with pale cytoplasm, a hypochromatic nucleus, and a small nucleolus. The hallmark finding in RDD is emperipolesis, which is the engulfment of viable lymphocytes inside the cytoplasm, unlike phagocytosis. These histiocytes also show diffuse cytoplasmic and nuclear staining for S100 and CD68 and negative staining for CD1a or CD207 [1]. Germline mutation in *SLC29A3* is notably associated with familial RDD patients; however, various kinase mutations, namely, *RAS*, *RAF*, and *MAP2K1* are also noted in RDD patients. The authors opine that genetic testing, if available, is preferable and may provide valuable insights into the disease and may bear therapeutic implications; however, histomorphology and immunohistochemistry help diagnose RDD [3]. The histopathological and immunohistochemical findings in our patient were consistent with the diagnosis of RDD and further genetic testing was not performed considering the cost of such investigations.

A variety of differential diagnoses and associated disorders should be considered and sequentially ruled out before making a diagnosis. Differential diagnoses and association of RDD with various diseases, including hemophagocytic lymphohistiocytosis (HLH), Langerhans cell histiocytosis, sinus histiocytosis, lymphomas, autoimmune diseases, immunoglobulin G4 disease (IgG4), is noted [11]. The absence of feature of hemophagocytosis and criteria used for the diagnosis of HLH ruled out HLH, the absence of increased IgG4-positive plasma cell ruled out IgG4 disease, negativity for CD1a and Langerin ruled out Langerhans cell histiocytosis, histomorphology and clinical findings ruled out sinus histiocytosis, and histomorphology along with characteristic immunostain positivity for CD68 and S100 along with clinical findings ruled out various lymphomatous neoplasms involving the region. No autoimmune disease responsible or associated with RDD was noted in the patient [3,11].

Most of the reported cases with intracardiac mass were managed with surgical excision and a few were started on corticosteroids [4]. Treatment for RDD is based on factors such as the site of lesion, availability of treatment, cost of treatment, and the risk versus benefit analysis. In our patient, surgery and corticosteroids were effective in the management of the disease; however, other modalities such as chemotherapy, immunotherapy, radiotherapy or targeted therapy, have shown promising results in relapsed and refractory cases of RDD. The use of accessory treatment modality in our patient was not considered because the patient improved post-surgery and with corticosteroid therapy and was disease-free on follow-up [3]. Nodal and cutaneous diseases have a favorable prognosis, whereas extranodal involvement has unpredictable outcomes.

## Conclusions

Tuberculosis remains the major cause of constrictive pericarditis in India. However, the majority of tuberculosis pericarditis cases present with pericardial effusion and a small fraction with constrictive pericarditis. In the absence of a histopathological diagnosis, this case potentially could have been mislabelled as a tubercular etiology. This case underscores the importance of tissue diagnosis, appropriate immunohistochemical stains, and relevant clinicoradiological findings in cases of constrictive pericarditis without an established diagnosis.
